# A systematic review investigating the cumulative incidence of chronic kidney disease in young adults with impaired glucose tolerance

**DOI:** 10.1186/s13643-015-0059-6

**Published:** 2015-05-13

**Authors:** Ferozkhan Jadhakhan, Tom Marshall, Paramjit Gill

**Affiliations:** Primary Care Clinical Sciences, University of Birmingham, Birmingham, B15 2TT UK

**Keywords:** Impaired glucose tolerance, Chronic kidney disease, Estimated glomerular filtration rate, Albumin creatinine ratio, Protein creatinine ratio, Serum creatinine, Creatinine clearance, Type 2 diabetes and young adults

## Abstract

**Background:**

It is known that risk of chronic kidney disease (CKD) is elevated in patients with diabetes mellitus but it is not clear whether the risk is also elevated with impaired glucose tolerance (IGT). If the risk is increased, it is not known if this is confined to people with IGT who progress to type 2 diabetes (T2DM). The purpose of this systematic review is to determine the relative risk of CKD in young adults (aged 18 to 40 years) with IGT (exposed group) compared to those without glycaemic abnormality (comparator group).

**Methods/Design:**

The following electronic databases will be systematically searched from inception to January 2015 for relevant studies: CINAHL, EMBASE, MEDLINE, PubMed, Cochrane libraries and grey literature. Two independent reviewers will screen search results, extract data, select studies for inclusion and assess their quality. Studies including young adults (aged 18 to 40 years) with IGT containing any of the following CKD markers will be included: estimated glomerular filtration rate (eGFR), albumin creatinine ratio (ACR), protein creatinine ratio (PCR), serum creatinine (SCr) and creatinine clearance (CrCl) levels. Studies at any time period after diagnosis of IGT and with any length of follow-up will be included. The proportion of IGT participants reporting each outcome will be documented. Relative risks (RR) and odds ratios (OR) will be extracted or calculated from raw data. If possible, study results will be combined in a meta-analysis.

**Discussion:**

The results of this comprehensive review will establish the evidence for the association between IGT and the risk of developing CKD in young adults (aged 18 to 40 years).

**Systematic review registration:**

PROSPERO CRD42014007081

**Electronic supplementary material:**

The online version of this article (doi:10.1186/s13643-015-0059-6) contains supplementary material, which is available to authorized users.

## Background

Chronic kidney disease (CKD) is a long-term condition which may lead to renal failure or in some cases to premature death if left undetected. CKD is characterised by the presence of kidney damage (albuminuria) and/or a gradual loss of kidney function (eGFR) over time [1]. The complex interrelationship of CKD and its associated comorbidities render this condition a substantial public health concern [2]. Furthermore, CKD is an independent risk factor for cardiovascular disease (CVD) [3]. Diabetes is the leading cause of chronic kidney disease and end stage renal disease (ESRD). CKD is present in 40 % of individuals with a history of diabetes compared to 15 % of individuals with no history of diabetes [4]. A recent study conducted in the UK showed that the risk of developing CKD (stages 3B, 4 and 5) in people with diabetes was eight times higher in women and over 12 times higher in men compared to those without diabetes [5].

On the other hand, impaired glucose tolerance (IGT) represents an intermediate state of normal glucose homeostasis and diabetic hyperglycaemia. Individuals who are classed as IGT have a blood sugar level raised beyond normal level, but it is not high enough to suggest full blown diabetes [6]. The prospective association of CKD with IGT has not been fully established. Cross-sectional data show some association that increasing blood glucose contributes to the decline in kidney function which may result in kidney disease and lead to kidney failure [7–9]. It is not clear whether the risk of CKD is also elevated in IGT. The current American Diabetes Association (ADA) definition of IGT is: fasting plasma glucose of 100–125 mg/dL (5.6–6.9 mmol/L), a blood glucose of 140–198 mg/dL (7.8–11.1 mmol/L) after a 2-h oral glucose tolerance test (OGTT) and a glycated haemoglobin (HbA1c) of 5.7–6.4 % (39–47 mmol/mol) [10]. Not everyone with IGT progresses to type 2 diabetes (T2DM); however, people with high glucose level are at greater risk of progressing to T2DM and subsequently develop CKD. A published analysis using data from the general practice research database (GPRD) estimated the prevalence of IGT/impaired fasting glucose (IFG) from a study population of approximately 2.8 million individuals registered with a GP practice between 1 January 2000 and April 2005. The annual prevalence of IGT/IFG during the 5-year study period increased from 17 cases per 100,000 individuals in 2001 to 31 cases per 100,000 individuals in 2005 [11].

### Risk of CKD amongst young adults (aged 18 to 40 years) with IGT

**Fig. 1:**
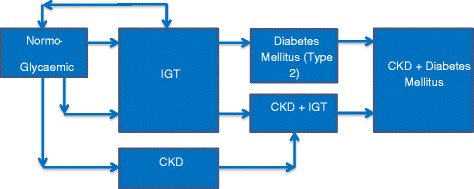
Progression of impaired glucose tolerance to type 2 diabetes or reversal to normoglycaemia and development of chronic kidney disease. IGT: impaired glucose tolerance; T2DM: type 2 diabetes; CKD: chronic kidney disease

In a cross-sectional study of adult aged ≥25 years, the prevalence of albuminuria, an early marker for the development of CKD, was 5.1 % with normal glucose tolerance, 11 % with IGT, 17.8 % newly diagnosed and 36.2 % known type 2 diabetes [12]. Similarly, in the data from the US National Health and Nutrition Examination Survey (NHANES) from 1999 to 2006, the prevalence of CKD was found to be 39.6 % in self-reported diagnosed diabetes, 41.7 % in previously undiagnosed diabetes (fasting plasma glucose [FPG] ≥126 mg/dL), 17.7 % in pre-diabetes (IGT ≥100 and <126 mg/dL) and 10.6 % in those without glycaemic abnormality [13]. These cross-sectional data are however subject to some limitations. It is unclear whether CKD precedes impaired glucose metabolism or vice versa (Fig. 1) and eGFR values were taken on only a single occasion.

### IGT trajectory and the development of CKD in young adults (aged 18 to 40 years)

Two epidemiological studies in Native American populations found evidence linking renal function to IGT. One found similar rates of decline in glomerular filtration rate (GFR) over 4 years in persons with IGT (14 % decline) and with newly diagnosed diabetes (18 % decline) [14]. The other found 15 % of the 934 non-diabetic participants had micro-albuminuria [15]. In contrast, in the data derived from the Framingham heart study offspring cohort (1991–1995) examining the development of CKD (eGFR <59 mL/min/1.73 m^2^ in women and <64 mL/min/1.73 m^2^ in men) after patients were given an oral glucose tolerance test and followed up for an average 7 years, the subsequent mean GFR at follow-up were: normoglycaemia (87 ml/min/1.73 m^2^), IGT (85 ml/min/1.73 m^2^), newly diagnosed diabetes (82 ml/min/1.73 m^2^) and know diabetes (78 ml/min/1.73 m^2^) [16]. As many studies have used a single determination of glycaemic status at baseline, it is not clear whether the risk of developing a CKD event is confined to people with IGT who progress to overt diabetes or whether the risk is still increased amongst people with IGT even if they never develop diabetes. Few studies have included young adults (aged 18 to 40 years) with IGT in their analyses. Studies that included individuals aged ≥18 years were cross-sectional by design and are subject to some limitations. This makes the determination of relative risk and incidence of CKD impossible. Also, it is unclear whether CKD preceded impaired glucose tolerance metabolism and eGFR values were measured on only a single occasion. Furthermore, some changes in the renal function (e.g. decrease glomerular filtration rate and increased albuminuria) may already be present in individuals with IGT before the onset of diabetes. Due to these limitations, it is inappropriate to extrapolate rates and relative risks to this cohort of individuals and in particular to primary care where majority of decisions for early prevention are made. It is therefore important to have a reliable estimate of the incidence of CKD in this cohort of individuals.

The overall purpose of this systematic review is to elucidate whether the presence of IGT is associated with an increased risk of CKD by comparing the risk of CKD in individuals with IGT to those without IGT.

### Aims

This systematic review will aim to:

- Determine the incidence of CKD in young adults (aged 18 to 40 years) with IGT.
- Determine the relative risk of developing CKD in young adults (aged 18 to 40 years) with IGT (exposed group) compared to those without glycaemic abnormality (comparator group).
- Determine whether the risk of CKD (compared to people without IGT) is increased in the subgroup of IGT patients who develop type 2 diabetes (T2DM) vs. IGT patients who do not develop T2DM. If data permits to examine whether there is any increasing CKD risk gradient across the three subgroups: unexposed group (people without IGT or diabetes), exposed group 1 (people with IGT but without diabetes) and exposed group 2 (people who have both IGT and diabetes).

## Methods/Design

Established guidelines for reviews will be used to inform the search strategy, for the selection of studies, assessment of risks of bias and reporting of results [17, 18].

### Eligibility criteria

#### Types of participants and comparison group

This review will include studies of participants aged 18 to 40 years without a diagnosis of type 1 and type 2 diabetes but with IGT, “Pre-diabetes” or “Pre-diabetic state”. Pre-diabetes can refer to either IGT or IFG [19], or metabolic syndrome where IGT is part of the metabolic syndrome. The comparison group will be either participants with normoglycaemia or diabetic participants. For the purpose of this review, IGT will be defined as a FPG <7 mmol/L or an OGTT ≥7.8 mmol/L and <11.1 mmol/L, or HbA1c of 6.0–6.4 % (42–47 mmol/mol) [20].

##### Participants and outcomes—cohort studies

This review will include any cohort studies where some participants are aged 18 to 40 years; results are reported separately in this age group with:

1. IGT but without a diagnosis of type 1 or type 2 diabetes compared to participants without glycaemic abnormality.
2. IGT but without a diagnosis of type 1 diabetes compared to participants with T2DM.

Exposed (cases with IGT) and comparator (without glycaemic abnormality or T2DM) groups must be free from CKD at baseline. Studies will be included that define chronic kidney disease by any of a number of measures. These include eGFR stages 3A, 3B, 4 and 5; albuminuria; albumin creatinine ratio (ACR ≥2.5 mg/mmol or ≥30 mg/g), protein creatinine ratio (PCR ≥45 mg/mmol or ≥300 mg/g), serum creatinine (SCr 1.0 mg/dL or ≥50 μmol/L) and creatinine clearance (CrCl ≥60 ml/min). Studies that report mean changes in continuous variables (e.g. eGFR) will be included and findings summarised separately. The incidence of CKD during follow-up must be reported or calculable in both exposed and comparator groups. Studies must either report relative risk for the above outcomes or the incidence of CKD must be reported or calculable from raw data in exposed and comparator groups allowing relative risk (RR) to be calculated. There will be no restriction on the length of participant follow-up.

##### Participants and outcomes—case control studies

This review will also include any case control studies where some cases are (aged 18 to 40 years) with an incident diagnosis of CKD (the outcome of interest) by any of the above definitions (eGFR stages 3A or higher; albuminuria; ACR, PCR, SCr and CrCl) and controls without a diagnosis of CKD. In the case control studies, the frequency of previous IGT (exposure to IGT) must be reported or calculable in both cases and controls and compared either to the frequency of normoglycaemia (unexposed) or to the frequency of diabetes (an alternative exposure). There will be no restriction on the length of time between CKD diagnosis and exposure in case–control studies. Studies must either report odds ratios or this must be calculable from the raw data extracted from the study.

### Exclusion criteria

Studies without a formal comparison group or control arm will be excluded from the review. Cross-sectional studies will be excluded because they do not distinguish between IGT diagnosed after CKD and IGT diagnosed before CKD. Randomised controlled trial including quasi-experimental designs will also be excluded.

### Sources of information

The following electronic databases will be systematically searched from inception to January 2015: MEDLINE, Cumulative Index to Nursing and Allied Health Literature (CINAHL), EMBASE, PubMed, Database of Abstracts of Reviews of Effects (DARE), Cochrane Database of Systematic Reviews (CDSR) and Trip Database. Furthermore, ongoing studies, scientific literature and abstract proceedings will be identified by searching the following databases: ClinicalTrials.gov, Cochrane Renal Group specialised register, Renal Registry Database, British Renal Society, Renal Association, American Society of Nephrology, World Congress of Nephrology, Diabetes UK Conference, Primary Care Diabetes Society Conference and Zetoc. A comprehensive search of the Conference Proceedings Citation Index (CPCI) will also be carried out. Search of these databases will span from January 2011 to January 2014 as it is likely that studies would have been completed and published. Grey literature databases, such as Grey Literature Report, OpenGrey, PubliCat and ScienceDaily.com will be examined. Google Scholar will also be explored; a scoping search revealed that the most pertinent articles were found in the first ten pages of the searches. Open access theses and dissertations will be retrieved from the ProQuest Dissertation Thesis Database and thesis.com. The Science Citation Index (SCI) will be used to scan and track study titles.

### Search

An optimal search strategy has been developed; an additional file shows this in more details (see Additional file 1) which focuses on the following key terms: chronic kidney disease, impaired glucose tolerance, type 2 diabetes and young adult.

### Study selection process

Two reviewers will independently review all titles and abstract of the search results in two phases. First, the retrieved titles and abstracts will be reviewed to identify relevant studies. Then, the full text of retrieved studies will be read to determine eligibility. Any discrepancies or difference in opinion will be resolved by consensus or by involving a third reviewer. An inclusion criteria checklist (Table 1) has been developed based on study eligibility criteria and piloted on five papers to check that studies are interpreted and classified appropriately. A PRISMA study flow diagram of included and excluded studies will be provided along with reasons for exclusion.

**Table 1:** Review eligibility criteria checklist

Study design	Cohort studies
	Case–control studies
Study characteristics	Full articles
	Conference proceedings
	Grey literature
	Theses/dissertations
	Other (please specify)
Participants	Studies where some participants are aged 18 to 40 years
	With IGT
	With pre-diabetes (can refer to either IGT or impaired fasting glucose (IFG)
	With metabolic syndrome (where IGT is part of metabolic syndrome)
	Free from CKD at baseline
Comparator	Participant with normoglycaemia
	Participants with diabetes
Outcome	Chronic kidney disease [eGFR stages: 3A, 3B, 4 and 5]
	Albuminuria
	ACR (albumin creatinine ratio ≥30 mg/mmol
	PCR (protein creatinine ratio ≥50 mg/mmol
	SCr (serum creatinine) data
	CrCl (creatinine clearance) data

### Data collection process

A data extraction form has been designed based on the Hayden et al. framework [21]; this is described in more details in an additional file (see Additional file 2). This form has been iteratively developed and will be pilot tested on known papers independently by two reviewers. The form has been designed to focus on population, comparator, outcome and study design. Data extraction will be conducted by one reviewer and checked by another for all studies identified through the screening phase. Errors in data extraction will be discussed and amended as required. For missing data, authors may be contacted for clarification. A Microsoft Excel sheet will be used to manage data extraction.

### Quality assessment

Quality assessment will be carried out by two reviewers independently. Difference in opinion will be resolved by consensus or by involving a third reviewer, if necessary. Study quality will be assessed by focusing on the following criteria: sampling, validated method to confirm outcome, attrition and analytical method. The form has been adapted from the Ottawa–Newcastle Scale (NOS) [22] to assess the quality of cohort and case–control studies to meet the specific needs of this systematic review. An additional file describes the stages and domain of this modified tool (see Additional file 3). The devised form has been assessed independently by two reviewers on known papers to ascertain its viability. Presentation of risk of bias assessment will be displayed in accordance with the Cochrane Collaboration recommendation. A composite score will not be provided; instead, a risk of bias of “yes” indicating low risk, “no”, high risk and “unclear” will be given to assess each domain [23]. A narrative summary of the overall quality of each study will be provided in a table. A critical appraisal of the study quality will be discussed along with the impact of the quality of studies on results. Studies rated poor quality will be excluded by conducting a sensitivity analysis.

### Data synthesis

Adjusted or unadjusted covariates, odds ratios (OR) or RR will be extracted or calculated from data provided. For cohort studies, cases (IGT) will be compared to normoglycaemia or type 2 diabetes. In case–control studies, participants with an incident diagnosis of CKD (cases) will be compared to participants without a diagnosis of CKD (controls). Data will be grouped and presented separately according to the study design. This will be presented in tables. If appropriate, cohort and case–control studies will be grouped separately and pooled estimates of the OR/RR for CKD compared to normoglycaemia and compared to diabetes will be calculated using STATA. Subgroup analyses may be performed if studies explore risks across groups for example: sex (female vs male), ethnicity (Black/Asians/other and White populations), age (≥18 ≤ 40) and adjustment for CKD comorbidities (e.g. hypertension, dyslipidaemia cardiovascular diseases and triglyceride). This will allow risk of CKD to be compared across these subgroups. Depending on the level of heterogeneity, both fixed and random effect models will be used as summary effect measure. A sensitivity analysis will be performed to examine the robustness of the meta-analysis under different assumptions: 1) will the result change if small sample size studies are removed, and 2) for studies which OR/RR were not provided and were calculated from raw data, will results of the pooled analysis change if these are removed. Risk ratio measures with 95 % confidence interval (CI) will be calculated for binary outcomes (e.g. incidence of CKD) or standardise mean difference with accompanying 95 % CI where continuous scales of measurements were used to identify CKD markers. If possible, CKD markers will be grouped into clinical stages based on GFR (3A, 3B, 4, and 5) and other indicators SCr, CrCl, ACR and PCR. Frequencies for each of the stages will be combined and pooled estimates calculated. The Cochrane Q test and I^2^ statistic with its 95 % (CI) will be used to assess heterogeneity across studies. Presence of publication bias and other reporting bias will be assessed with the use of the inverted funnel plot technique and the Egger statistic will be used to test for bias. The GRADE framework may be used to assess the quality and inconsistency between studies, risk of bias including publication bias, precision of results and applicability of results to the study population [24].

## Discussion

This systematic review will synthesise research evidence to establish whether the risk of developing CKD is relatively high in young adults with IGT and gather information on IGT progression to T2DM and development of CKD in this age group. Strengths and limitations will be highlighted in the identified evidence. Strength of observational data may include large sample size, high rate of follow-up and frequency of CKD more likely to be representative of the population at risk. Limitations may include the quality of data extracted which may not allow studies to be combined in a meta-analysis. This may be overcome by presenting the findings in a descriptive manner. This review was conducted in collaboration with an experienced librarian who helped appraise the search criteria, refine the keywords and MeSH terms and identify appropriate database(s). Screening and data extraction will be conducted by three reviewers employing a data extraction form which has been reviewed and pretested. Furthermore, this review is not limited to the English language. To the best of our knowledge, no reviews have been published exploring the study question; however, if a review addressing a similar question is published, it will be incorporated in this review and added in a meta-analysis if feasible.

### Implications of results

This systematic review will provide an updated and quantifiable estimate of the risk of CKD in young adults with IGT compared to those without IGT and compared to young adults with diabetes. If it is found that the frequency of CKD is elevated in young adults (aged 18 to 40 years) with IGT, then the management is likely to be inadequate. This review may inform policy change and implementation of preventative measures in this age group. Furthermore, the systematic search will identify where future research is required. For instance, this review may inform a prognostic study which may be useful in understanding the course and factors associated with CKD development.

## Additional files

Additional file 1: **Electronic search strategy used to conduct comprehensive literature search.** Electronic search of Medline for CKD outcomes.

Additional file 2: **Data extraction form adapted from Hayden et al. [** 21 **].** Framework for prognostic studies. Tools and domains to assess risk of bias at different stages of data extraction.

Additional file 3: **Quality assessment form adapted from the Ottawa-Newcastle scale (NOS) for assessing non-randomised studies.** Quality assessment form adapted to reflect the nature of cohort and case–control studies related to CKD outcomes.

## Abbreviations

CKD: Chronic kidney disease
IGT: Impaired glucose tolerance
T2DM: Type 2 diabetes
eGFR: Estimated glomerular filtration rate
ACR: Albumin creatinine ratio
PCR: Protein creatinine ratio
SCr: Serum creatinine
CrCl: Creatinine clearance
RR: Relative risk
OR: Odds ratio
CVD: Cardiovascular disease
OGTT: Oral glucose tolerance test
HbA1c: Glycated haemoglobin
IFG: Impaired fasting glucose
ESRD: End stage renal disease
NHANES: National Health and Nutrition Examination Survey
FPG: Fasting plasma glucose
BMI: Body mass index
DARE: Database of abstracts of reviews of effects
CDSR: Cochrane database of systematic reviews
CPCI: Conference Proceedings Citation Index
SCI: Science Citation Index
CINAHL: Cumulative Index for Nursing and Allied Health Literature
UK: United Kingdom
PROSPERO: Prospective Registering of Systematic Reviews
GRADE: Grading of recommendations assessment, development and evaluation

## References

[1] Levey AS, Coresh J, Balk E, Kausz AT, Levin A, Steffes MW (2003). National Kidney Foundation practice guidelines for chronic kidney disease: evaluation, classification, and stratification. Ann Intern Med..

[2] Coresh J, Astor BC, Greene T, Eknoyan G, Levey GS (2003). Prevalence of chronic kidney disease and decreased kidney function in the adult US population: Third National Health and Nutrition Examination Survey. Am J Kidney Dis..

[3] Weiner DE, Tighouart H, Amin MG, Stark PC, MacLeod B, Griffith JL (2004). Chronic kidney disease as a risk factor for cardiovascular disease and all-cause mortality: a pooled analysis of community-based studies. J Am Soc Nephrol..

[4] Collins AJ, Foley RN, Chavers B, Gilberton D, Herzog C, Johansen K (2012). United States renal data system 2011 annual data report: atlas of chronic kidney disease & end-stage renal disease in the United States. Am J Kidney Dis.

[5] Hippisley-Cox J, Coupland C (2010). Predicting the risk of chronic kidney disease in men and women in England and Wales: prospective derivation and external validation of the QKidney Scores. BMC Fam Pract..

[6] Unwin N, Shaw J, Zimmet P, Alberti KG (2002). Impaired glucose tolerance and impaired fasting glycaemia: the current status on definition and intervention. Diabet Med..

[7] Sechi LA, Catena C, Zingaro L, Melis A, DeMarchi S (2002). Abnormalities of glucose metabolism in patients with early renal failure. Diabetes..

[8] Kubo A, Kiyohara Y, Kato I, Iwamoto H, Nakayama K, Hirakata H (1999). Effect of hyperinsulinemia on renal function in a general Japanese population. Kidney Int..

[9] Chen J, Muntner P, Hamm LL, Fonseca V, Batuman V, Whelton PK (2003). Insulin resistance and risk of chronic kidney disease in nondiabetic US adults. J Am Soc Nephrol..

[10] American Diabetes Association (2010). Diagnosis and classification of diabetes mellitus. Diabetes Care.

[11] Gillett M, Royle P, Snaith A, Scotland G, Poobalan A, Imamura M (2012). Non-pharmacological interventions to reduce the risk of diabetes in people with impaired glucose regulation: a systematic review and economic evaluation. Health Technol Assess..

[12] Tapp RJ, Shaw JE, Zimmet PZ, Balkau B, Chadban SJ, Tonkin AM (2004). Albuminuria is evident in the early stages of diabetes onset: results from the Australian Diabetes, Obesity, and Lifestyle Study (AusDiab). Am J Kidney Dis..

[13] Plantinga LC, Crews DC, Coresh J, Miller ER, Saran R, Yee J (2010). Prevalence of chronic kidney disease in US adults with undiagnosed diabetes or prediabetes. Clin J Am Soc Nephrol..

[14] Nelson RG, Bennett PH, Beck GJ, Tan M, Knowler WC, Mitch WE (1996). Development and progression of renal disease in Pima Indians with noninsulin-dependent diabetes mellitus: Diabetic Renal Disease Study Group. N Engl J Med..

[15] Hoehner CM, Greenlund KJ, Rith-Najarian S, Casper ML, McClellan WM (2002). Association of the insulin resistance syndrome and microalbuminuria among nondiabetic Native Americans: the Inter-Tribal Heart Project. J Am Soc Nephrol..

[16] Fox CS, Larson MG, Leip EP, Meigs JB, Wilson PW, Levy D (2005). Glycemic status and development of kidney disease: the Framingham Heart Study. Diabetes Care..

[17] Liberati A, Altman DG, Tetzlaff J, Mulrow C, Gøtzsche PC, Ioannidis JPA (2009). The PRISMA statement for reporting systematic reviews and meta-analyses of studies that evaluate healthcare interventions: explanation and elaboration. J Clin Epidemiol..

[18] Stroup DF, Berlin JA, Morton SC, Olkin I, Williamson GD, Rennie D (2000). Meta-analysis of observational studies in epidemiology: a proposal for reporting: meta-analysis of observational studies in epidemiology (MOOSE) group. JAMA..

[19] Buysschaert M, Bergman M (2011). Definition of prediabetes. Med Clin North Am.

[20] Nathan DM, Balkau B, Bonora E, Borch-Johnsen K, Buse JB, Colagiuri S (2009). International Expert Committee report on the role of the A1C assay in the diagnosis of diabetes. Diabetes Care..

[21] Hayden JA, Côté P, Bombardier C (2006). Evaluation of the quality of prognosis studies in systematic reviews. Ann Intern Med..

[22] Hartling L, Milne A, Hamm MP, Vandermeer B, Ansari M, Tsertsvadze A (2013). Testing the Newcastle Ottawa Scale showed low reliability between individual reviewers. J Clin Epidemiol..

[23] Higgins JP, Altman DG, Gotzsche PC, Juni P, Moher D, Oxman AD (2011). The Cochrane Collaboration’s tool for assessing risk of bias in randomised trials. BMJ..

[24] Guyatt G, Oxman AD, Akl EA, Kunz R, Vist G, Brozek J (2011). GRADE guidelines: 1. introduction-GRADE evidence profiles and summary of findings tables. J Clin Epidemiol.

