# Supplementary material for: A systematic review investigating the cumulative incidence of chronic kidney disease in young adults with impaired glucose tolerance
Source: Syst Rev. 2015 May 13;4:69. doi: 10.1186/s13643-015-0059-6 (PMC4433064; doi:10.1186/s13643-015-0059-6)
Supplement: Supplementary file 1 — Electronic search strategy used to conduct comprehensive literature search. Electronic search of Medline for CKD outcomes. [file 13643_2015_59_MOESM1_ESM.pdf]

Additional file 1

Electronic search of Medline for CKD outcomes

Count	Searches	Results
1	exp Renal Insufficiency Chronic/	84840
2	chronic kidney disease.mp.	18247
3	chronic kidney disease$.mp.	18745
4	exp Kidney Failure, Chronic/	78448
5	chronic kidney failure.mp.	975
6	chronic kidney failure$.mp.	976
7	chronic renal failure.mp.	20307
8	chronic renal failure$.mp.	20319
9	end stage kidney disease.mp.	997
10	end stage kidney disease$.mp.	1005
11	esrd.mp.	9767
12	esrd$.mp.	9769
13	chronic kidney insufficiency.mp.	195
14	chronic kidney insufficiency$.mp.	195
15	end stage renal disease.mp.	19454
16	end stage renal disease$.mp.	19625
17	end stage renal failure.mp.	4888
18	end stage renal failure$.mp.	4891
19	kidney failure.mp.	81179
20	kidney failure$.mp.	81828
21	renal insufficiency/	10919
22	renal failure.mp.	71184
23	renal failure$.mp.	71271
24	kidney insufficiency.mp.	577
25	kidney insufficiency$.mp.	577
26	exp renal dialysis/	93204
27	renal dialysis.mp.	74276
28	renal dialysis$.mp.	74276
29	extracorporeal dialysis.mp.	161
30	extracorporeal dialysis$.mp.	162
31	hemodialysis.mp.	47033
32	hemodialysis$.mp.	47039
33	haemodialysis.mp.	11565
34	haemodialysis$.mp.	11576
35	exp peritoneal dialysis/	22761
36	peritoneal dialysis.mp.	26208
37	peritoneal dialysis$.mp.	26210
38	renal disease.mp.	39428
39	renal disease$.mp.	43692
40	exp kidney diseases/	414228
41	kidney disease.mp.	30990

Count	Searches	Results
42	kidney disease$.mp.	103750
43	nephropathy.mp.	37719
44	nephropathy$.mp.	37721
45	exp diabetic nephropathies/	19707
46	diabetic nephropathy.mp.	11909
47	diabetic nephropathy$.mp.	11909
48	exp kidney transplantation/	79900
49	kidney transplantation.mp.	81478
50	kidney transplantation$.mp.	81539
51	renal transplant.mp.	18888
52	renal transplant$.mp.	36596
53	exp dialysis/	22124
54	dialysis.mp.	132315
55	dialysis$.mp.	132329
56	exp renal insufficiency/	127094
57	renal insufficiency.mp.	33560
58	renal insufficiency$.mp.	33571
59	EGFR.mp.	27296
60	EGFR$.mp.	27669
61	exp glomerular filtration rate/	33096
62	glomerular filtration rate.mp.	42765
63	glomerular filtration rate$.mp.	43111
64	exp creatinine/	46991
65	creatinine.mp.	95418
66	creatinine$.mp.	95673
67	serum creatinine.mp.	26670
68	serum creatinine$.mp.	26781
69	serum creatinine clearance.mp.	53
70	serum creatinine clearance$.mp.	58
71	exp albuminuria/	12045
72	albuminuria$.mp.	14614
73	exp proteinuria/	32445
74	proteinuria.mp.	36905
75	proteinuria$.mp.	36921
76	1 or 2 or 3 or 4 or 5 or 6 or 7 or 8 or 9 or 10 or 11 or 12 or 13 or 14 or 15 or 16 or 17 or 18 or 19 or 20 or 21 or 22 or 23 or 24 or 25 or 26 or 27 or 28 or 29 or 30 or 31 or 32 or 33 or 34 or 35 or 36 or 37 or 38 or 39 or 40 or 41 or 42 or 43 or 44 or 45 or 46 or 47 or 48 or 49 or 50 or 51 or 52 or 53 or 54 or 55 or 56 or 57 or 58 or 59 or 60 or 61 or 62 or 63 or 64 or 65 or 66 or 67 or 68 or 69 or 70 or 71 or 72 or 73 or 74 or 75	683333
77	exp diabetes mellitus, type 2/	87630
78	diabetes mellitus type 2.mp.	87782
79	type 2 diabetes.mp.	60366
80	type 2 diabetes$.mp.	60434

Count	Searches	Results
81	niddm.mp.	6673
82	niddm$.mp.	6704
83	exp diabetes insipidus/	7011
84	diabetes insipidus.mp.	8589
85	diabetes insipidus$.mp.	8589
86	77 or 78 or 79 or 80 or 81 or 82 or 83 or 84 or 85	113193
87	exp glucose intolerance/	6360
88	impaired glucose tolerance.mp.	8065
89	impaired glucose tolerance$.mp.	8066
90	glucose intolerance.mp.	11300
91	glucose intolerance$.mp.	11302
92	exp prediabetic state/	3852
93	prediabetes.mp.	1633
94	prediabetic state.mp.	4004
95	prediabetic state$.mp.	4053
96	exp blood glucose/	130285
97	blood glucose.mp.	147811
98	blood glucose$.mp.	147841
99	glucose metabolism.mp.	23463
100	glucose metabolism$.mp.	23713
101	exp glucose tolerance test/	29397
102	glucose tolerance test.mp.	33799
103	glucose tolerance test$.mp.	34976
104	OGTT.mp.	5382
105	OGTT$.mp.	5481
106	exp Hyperglycemia/	26636
107	hyperglycemia.mp.	37642
108	hyperglycemia$.mp.	37682
109	hyperglycaemia.mp.	6827
110	hyperglycaemia$.mp.	6840
111	impaired fasting glucose.mp.	2336
112	impaired fasting glucose$.mp.	2336
113	postprandial hyperglycemia.mp.	911
114	postprandial hyperglycaemia.mp.	283
115	exp hemoglobin a, glycosylated/	23254
116	hemoglobin a, glycosylated.mp.	23255
117	hemoglobin a, glycosylated$.mp.	23255
118	Haemoglobin a, glycosylated.mp.	1
119	HbA1c.mp.	13678
120	HbA1c$.mp.	13741
121	glycemic abnormality.mp.	7
122	Glycaemic abnormality.mp.	0
123	Fasting plasma glucose.mp.	7250

Count	Searches	Results
124	Fasting plasma glucose$.mp.	7259
125	87 or 88 or 89 or 90 or 91 or 92 or 93 or 94 or 95 or 96 or 97 or 98 or 99 or 100 or 101 or 102 or 103 or 104 or 105 or 106 or 107 or 108 or 109 or 110 or 111 or 112 or 113 or 114 or 115 or 116 or 117 or 118 or 119 or 120 or 121 or 122 or 123 or 124	223393
126	76 and 86 and 125	4367
127	exp cohort studies/	1387399
128	cohort$.tw.	263907
129	controlled clinical trial.pt.	88411
130	epidemiologic methods/	29569
131	exp case-control studies/	688110
132	(case$ and control$).tw.	311313
133	127 or 128 or 129 or 130 or 131 or 132	1909067
134	cohort studies/	170386
135	longitudinal studies/	87299
136	follow-up studies/	504746
137	prospective studies/	376036
138	retrospective studies/	512412
139	cohort.ti,ab.	239106
140	longitudinal.ti,ab.	132074
141	prospective.ti,ab.	339467
142	retrospective.ti,ab.	264289
143	Case-Control Studies/	188791
144	Control Groups/	1435
145	Matched-Pair Analysis/	4154
146	retrospective studies/	512412
147	((case* adj5 control*) or (case adj3 comparison*) or control group*).ti,ab.	374925
148	127 or 128 or 129 or 130 or 131 or 132 or 133 or 134 or 135 or 136 or 137 or 138 or 139 or 140 or 141 or 142 or 143 or 144 or 145 or 146 or 147	2266708
149	126 and 148	1552
150	Remove duplicates from 149	1549
