# Supplementary material for: A systematic review investigating the cumulative incidence of chronic kidney disease in young adults with impaired glucose tolerance
Source: Syst Rev. 2015 May 13;4:69. doi: 10.1186/s13643-015-0059-6 (PMC4433064; doi:10.1186/s13643-015-0059-6)
Supplement: Supplementary file 2 — Data extraction form adapted from Hayden et al. [ 21 ]. Framework for prognostic studies. Tools and domains to assess risk of bias at different stages of data extraction. [file 13643_2015_59_MOESM2_ESM.pdf]

Additional file 2

Data extraction form adapted from Hayden et al Framework

Abbreviation

IGT	Impaired Glucose Tolerance
CKD	Chronic Kidney Disease
T2DM	Type 2 Diabetes
SCr	Serum Creatinine
CrCl	Creatinine Clearance
ESRD	End Stage Renal Disease
ACR	Albumin Creatinine Ratio
PCR	Protein Creatinine Ratio
EX	Excluded
NR	Not Reported
eGFR	Estimated Glomerular Filtration Rate

Eligibility criteria for title and abstract screening phase

Study design	Assessment	Comment
Is it:<br>[1] A cohort study (Prospective or Retrospective)<br>[2] A case-control or nested case-control study	Yes<br>No<br>Unclear	
**Population**		
[1] Were patients diagnosed with IGT?<br>[2] Were patients followed up for T2DM<br><br>NB: Please answer Yes if IGT diagnosed in a sub group	Yes<br>No<br>Unclear	
Are patients aged (18-40) years<br><br>NB: Please answer Yes if mixed age population	Yes<br>No<br>Unclear	

<table>
<tr><th>Study design</th><th>Assessment</th><th>Comment</th></tr>
<tr><td>Outcomes</td><td></td><td></td></tr>
<tr><td>Did the study report any of the following outcome:
<table>
<tr><td>Stage</td><td>eGFR (ml/min/1.73m^2)</td></tr>
<tr><td>3A</td><td>45-59</td></tr>
<tr><td>3B</td><td>30-44</td></tr>
<tr><td>4</td><td>15-29</td></tr>
<tr><td>5</td><td><15</td></tr>
</table>
ESRD (stage 5)
Albuminuria
ACR - >30mg/mmol
PCR - >45mg/mmol
SCr measures
CrCl measures</td><td>Yes
No
Unclear</td><td></td></tr>
<tr><td>Follow up</td><td></td><td></td></tr>
<tr><td>Were the patients followed up and adequate measures taken

NB: Please answer Yes if adequate measure were taken and key characteristics described</td><td>Yes
No
Unclear</td><td></td></tr>
<tr><td>Final decision (please tick)</td><td>Include
Exclude
Unclear</td><td></td></tr>
</table>

Exclusion criteria

**Reasons for exclusion of study from review** (please circle where appropriate)	
Methods	Not a cohort/case-control study
Patients	No IGT/no T2DM follow up/T2DM or Type 1 diabetes diagnosis /CKD /no (18 to 40 years) age group
Outcomes	No relevant outcomes assessed<br>No data for relevant subgroup extractable
Follow up period	No follow up
Other	Duplicate publication<br>Other

Inclusion criteria

**Specific inclusion criteria** (please include if answer is Yes to all questions below)	
**Eligibility criteria**	
Satisfaction of eligibility criteria	Yes<br>No<br>Unclear
**Effect sizes**	
Is there sufficient reporting of statistics or data to calculate effect sizes	Yes<br>No<br>Unclear

Organisation

**Organisational aspect**		**Exclude**		**Include**
**Reviewer/date:**		**Checked by:**		
Author/Year				
Journal/Source				
Country of origin				
Publication type	Full text/Abstract/Book chapter/progress report/<br>Other – please specify			
Fate	Decision pending/Check references/Use for discussion/EX without listing/EX with listing<br>Other – please specify			
Notes				

Study characteristics

<table>
<tr><th colspan="2">General study characteristics (please circle where appropriate)</th></tr>
<tr><td>Location of study</td><td></td></tr>
<tr><td>Study aims</td><td>Reported/NR</td></tr>
<tr><td>Dates of recruitment</td><td>From ________ to _________
Median (range): #
Mean:#</td></tr>
<tr><td>Length of follow up of CKD outcome + length of follow up of study</td><td>From ________ to _________
Median (range): #
Mean:#</td></tr>
<tr><td>Outcomes assessed</td><td>Did the study report any of the following outcome:
<table>
<tr><td>Stage</td><td>eGFR (ml/min/1.73m^2)</td></tr>
<tr><td>3A</td><td>45-59</td></tr>
<tr><td>3B</td><td>30-44</td></tr>
<tr><td>4</td><td>15-29</td></tr>
<tr><td>5</td><td><15</td></tr>
</table>
End Stage Renal Disease (stage 5)
Albumin creatinine ratio - >30mg/mmol
Protein creatinine ratio - >45mg/mmol
Serum creatinine measures
Creatinine clearance measures
Other (please specify):</td></tr>
<tr><td>Outcome definition</td><td></td></tr>
<tr><td>Relationship between outcome and relevant factor</td><td>Is the relationship statistically significant?
Yes/No
RR/OR: #

If No, is it due to
Low powered or inconclusive study/A true negative study</td></tr>
<tr><td>Power calculation</td><td>Yes/No/Not reported

Calculated sample size: #
Sample size achieved: Yes/No</td></tr>
<tr><td></td><td></td></tr>
<tr><td>Funding</td><td>Unclear
NR
Please state where reported</td></tr>
<tr><td>Conflict of interest statement</td><td>Yes/No/NR</td></tr>
</table>

<table>
<tr><th colspan="4">Baseline characteristics of patients (please circle where appropriate)</th></tr>
<tr><td></td><td>Exposure</td><td>Control</td><td>Notes: any relationship with outcomes? Yes/No/NR
If Yes please state if statistically significant and RR/OR values</td></tr>
<tr><td colspan="4">Overall comment: Significant/In significant</td></tr>
<tr><td>Number of patients</td><td></td><td></td><td></td></tr>
<tr><td>Age range (if reported)
Mean</td><td></td><td></td><td></td></tr>
<tr><td>Ethnicity
No%</td><td></td><td></td><td></td></tr>
<tr><td>Gender
No%</td><td>Male:
Female:</td><td>Male:
Female:</td><td></td></tr>
<tr><td>No of patients screened for IGT</td><td></td><td></td><td></td></tr>
<tr><td>No of patients recruited</td><td></td><td></td><td></td></tr>
<tr><td>No of patients allocated</td><td></td><td></td><td></td></tr>
<tr><td>No of patients evaluated</td><td></td><td></td><td></td></tr>
<tr><td>No of dropouts</td><td></td><td></td><td></td></tr>
<tr><td>Reasons for dropouts</td><td></td><td></td><td></td></tr>
<tr><td></td><td></td><td></td><td></td></tr>
<tr><td>Number of protocol violations</td><td></td><td></td><td></td></tr>
<tr><td></td><td colspan="3"></td></tr>
<tr><td>Definition of IGT
<table>
<tr><td>Fasting plasma glucose</td><td>6.1-6.9 mmol/L</td></tr>
<tr><td>Oral Glucose Tolerance test (2h value)</td><td>7.8-11.0 mmol/L</td></tr>
<tr><td>HbA1c</td><td>42-47 mmol/mol</td></tr>
</table>
Please circle all that applies and list all</td><td></td><td></td><td></td></tr>
<tr><td>Status of patient at recruitment

Any treatment for any comorbidities

If treated:
Please state

What treatment

Duration</td><td></td><td></td><td></td></tr>
<tr><td>Adverse event?
Yes/No

If Yes please state</td><td></td><td></td><td></td></tr>
</table>

Observational study characteristics (please circle where appropriate)	
Sample size	
Number of excluded patients	
Recruitment method	
Type of observational study	Cohort studies (retrospective/prospective)<br>Case-control studies/nested case-control
Are group comparable?	Yes/No<br><br>If No, please specify
Any confounders?	Yes/No<br><br>If No, please specify
Analysis	
Drop outs stated	Yes/No<br><br>If Yes: # in each group

Outcome details

The following table have to be copied for every relevant outcome assessed *(please fill out fields only where applicable)*

**Outcomes assessed (please state where relevant)**	
Definition of each outcome	
Time of assessment of each outcome (post IGT)	
Timing of assessment	
Length of follow up for each outcome	
Method of measurement	
No of patients evaluated for each outcome, as stated above	

**Methodological quality summary for observational studies**					
Reviewer/Date:			Checked by:		
Contents (please refer to tables below for guidance	Yes	Partly	No	Unsure	Comments
Study participation					
Study attrition					
Measurement of prognostic factors					
Measurement and controlling for confounding variables					
Measurement of outcomes					
Analysis approach					
Summarised validity	Low risk of bias		Moderate risk of bias		High risk of bias
Remarks:					
