# Supplementary material for: A systematic review investigating the cumulative incidence of chronic kidney disease in young adults with impaired glucose tolerance
Source: Syst Rev. 2015 May 13;4:69. doi: 10.1186/s13643-015-0059-6 (PMC4433064; doi:10.1186/s13643-015-0059-6)
Supplement: Supplementary file 3 — Quality assessment form adapted from the Ottawa-Newcastle scale (NOS) for assessing non-randomised studies. Quality assessment form adapted to reflect the nature of cohort and case–control studies related to CKD outcomes. [file 13643_2015_59_MOESM3_ESM.pdf]

Additional file 3

Quality assessment form adapted from the Ottawa-Newcastle scale (NOS) for assessing non-randomised studies

<table>
<tr><th></th><th></th><th>Yes/No/Unclear</th></tr>
<tr><td>Selection of participants</td><td>[1] Was the inclusion/exclusion clearly described?
(for example, age, diagnosis status, IGT)
[2] Was inclusion/exclusion assessed using valid and reliable measures? (for example, if there are important inclusion/exclusion criteria that are not directly related to exposure and outcome and for which the accuracy of measurement may need scrutiny, e.g age)
[3] Was recruitment strategy clearly described?
[4] Did the investigators ensure that the exposed/unexposed group were comparable (for example, did they use stratification, matching or propensity Score)</td><td></td></tr>
<tr><td>Adequate description of study population</td><td>[1] Was study population well characterised?
Age
Sex
Ethnicity
Suitable definition of IGT</td><td></td></tr>
<tr><td>Validated method for ascertaining exposure</td><td>[1] Was the method used to ascertain exposure clearly defined?
[2] Was a valid and reliable measure used to ascertain exposure?
(For example what diagnostic test was used to confirm IGT)<table><tr><td>Fasting Plasma Glucose</td><td>6.1 – 6.9 mmol/L</td></tr><tr><td></td><td></td></tr><tr><td>Oral Glucose Tolerance Test (2h value)</td><td>7.8 – 11.0 mmol/L</td></tr><tr><td>HbA1c</td><td>42 – 47 mmol/mol</td></tr></table></td><td></td></tr>
<tr><td>Validated method to confirm outcome</td><td>[1] Was valid and reliable measures used to ascertain outcome? For example<table><tr><td>Stage</td><td>eGFR (ml/min/1.73m^2)</td></tr><tr><td>1</td><td>≥90</td></tr><tr><td>2</td><td>60-89</td></tr><tr><td>3A</td><td>45-59</td></tr><tr><td>3B</td><td>30-44</td></tr><tr><td>4</td><td>15-29</td></tr><tr><td>5</td><td><15</td></tr></table>ACR - >30mg/mmol
PCR - >45mg/mmol
SCr measures
CrCl measures</td><td></td></tr>
<tr><td>Adequate follow up period</td><td>[1] Was follow up long enough for the outcome to occur?
[2] Was the follow up period the same across all groups?
[3] Were differences in follow-up adjusted for using statistical techniques, e.g., survival analysis?</td><td></td></tr>
<tr><td>Completeness of follow-up (Attrition)</td><td>[1] Were drop-out rates and reasons for drop-out similar across exposed and unexposed?
[2] Were numbers of dropouts/withdrawals documented at</td><td></td></tr>
</table>

		Yes/No/Unclear
	each time point?	
Analysis controls for confounding	[1] Does the study identify and control for important confounding variables and effect modifiers?	
Sample size calculated	[1] Is the sample size adequate?<br>[2] Did the study describe how the sample size was calculated?<br>• Did the investigators conduct a power analysis to determine the adequacy of study group sizes for the outcome of interest?<br>• Was the sample size large enough to detect differences in event or a significant OR/RR between groups?<br>(For example, OR/RR increases of ≥1.5 or decrease of ≥0.67 between groups).	
Analytical methods appropriate	[1] Was the kind of analysis done appropriate for the kind of outcome data? For example,<br>• Dichotomous – logistic regression, survival<br>• Categorical – mixed model for categorical outcomes<br>• Continuous – Mixed model, ANCOVA<br>[2] Was loss to follow up accounted for in the analysis<br>(For example, through sensitivity analysis)	
